# Factors affecting the experience of procedural and postoperative pain in children and adolescents - a systematic review

**DOI:** 10.1186/s12903-026-10060-8

**Published:** 2026-10-01

**Authors:** Rikard Roxner, Henrik Berlin, Helena Domeij, Martina Vall, Susanne Brogårdh-Roth, Lubna Alward, Sofia Petrén, Liselotte Paulsson, Gunilla Klingberg

**Affiliations:** 1 Department of Pediatric Dentistry, Faculty of Odontology, Malmö University, Malmö, SE-205 06 Sweden; 2 Health Technology Assessment – Odontology, Faculty of Odontology, Malmö University, Malmö, Sweden; 3 Malmö University Library, Malmö University, Malmö, Sweden; 4 Department of Orthodontics, Faculty of Odontology, Malmö University, Malmö, Sweden

**Keywords:** Procedural pain, Postoperative pain, Exposure, Systematic review, Children, Adolescents

## Abstract

**Aim:**

To conduct a systematic review of factors contributing to 3–18-year-old children’s and adolescents’ experiences of procedural and postoperative pain.

**Materials and methods:**

A PECO protocol was constructed. Searches were conducted in PubMed, CINAHL, PsycINFO, and Web of Science, up to January 2026. The researchers (reading in pairs) assessed identified studies independently, according to the defined inclusion and exclusion criteria. The report followed the PRISMA statement.

**Results:**

Ten thousand, seven hundred twenty records were screened, 109 reports read in full text, and finally 18 articles were evaluated for risk of bias. Eight studies based on seven study populations were judged to have low or moderate overall risk of bias and were included in the review. These studies investigated whether the following exposure factors can influence pain experiences in children and adolescents: sex, age, dental anxiety, personal traits, parental anxiety, previous painful experiences, and genotype. For the factors sex, age and dental fear and anxiety, which were studied in at least two studies, synthesis without meta-analysis (SWiM) were conducted, and the certainty of evidence in the findings was determined using GRADE. The results show that there is a low certainty of evidence that female adolescents (12–18 years) rate procedural or postoperative pain higher than males. Furthermore, there is a very low certainty of evidence regarding the findings concerning age and dental anxiety.

Consequently, the findings should be interpreted with caution. Ethical concerns were highlighted, as three of the eight included studies lacked complete information on ethical review and informed consent procedures.

**Conclusions:**

Although several factors may be associated with children’s and adolescents’ pain experiences in conjunction with dental and medical treatment, the available evidence remains limited and the present review’s results should be interpreted with caution. The results imply that pain may be multifactorial which calls for individualised care.

**Trial registration:**

The study protocol was registered in PROSPERO (CRD42022342566), available at https://www.crd.york.ac.uk/prospero/display_record.php?ID=CRD42022342566.

**Supplementary Information:**

The online version contains supplementary material available at https://doi.org/10.1186/s12903-026-10060-8.

## Introduction

Pain is a complex, subjective [1], and common experience among children and adolescents in different dental and medical care settings [2–5]. It is influenced by biological, psychological, and societal factors [1]. Previous research has suggested that age [6–8], sex [9], dental anxiety [10], and parental trait anxiety [11, 12] are among the factors that influence the pain experience in children. However, current scientific evidence regarding factors influencing pain experienced by children and adolescents in clinical settings remains limited. This is problematic, as pain in conjunction with dental and medical procedures is not only distressing and uncomfortable, but may also lead to dental fear and anxiety [10], procedural fear [13], and avoidance behaviours [13–16]. The potentially detrimental consequences of pain highlight the importance of identifying individuals at risk of experiencing pain.

To date, no systematic review has synthesised evidence regarding factors influencing children’s and adolescents’ experiences of procedural or postoperative pain in medical and dental settings. Such a systematic review is necessary to map existing evidence, identify possible evidence gaps, and to inform both clinical practice and future research. This systematic review aimed at assessing present research on factors affecting children’s and adolescents’ experiences of self-reported procedural and postoperative pain in conjunction with dental or medical procedures. The research question was: What factors contribute to experiences of procedural and postoperative pain in children and adolescents, aged 3–18 years?

This report followed the PRISMA 2020 statement [17].

## Materials and methods

### Protocol and registration

The study protocol was registered in PROSPERO (CRD42022342566), available at https://www.crd.york.ac.uk/prospero/display_record.php?ID=CRD42022342566.

### Eligibility criteria

#### Inclusion criteria

The PECO framework (Population, Exposure, Comparator, and Outcome) was used to structure the research question, and inclusion criteria [18]. Studies were considered eligible if they met the following criteria:

- Population: Children and adolescents, aged 3 to 18 years, undergoing or having undergone dental (e.g., filling therapy, pulp therapy, tooth extraction) or medical procedures (e.g., vaccination, venipuncture, surgical procedures).
- Exposure: Age, sex, previous pain experiences, general or dental anxiety, temperamental factors, factors related to family situation or parental anxiety, other biological factors.
- Comparator: Exposure or no exposure.
- Outcome: Self-reported procedural pain or postoperative pain measured with any of the following tools: Visual Analogue Scale (VAS) [19], Numeric Rating Scale (NRS) [20], Oucher Pain Scale [21], Wong-Baker FACES^®^ Pain Rating Scale [22], Coloured Analogue Scale (CAS) [23], and Faces Pain Scale-Revised (FPS-R) [24].

Study design: randomised controlled trials, non-randomised studies, observational studies, studies using qualitative methods, and systematic reviews.

Language: English, Danish, Norwegian, and Swedish.

Time frame: date of publication up to January 2026.

#### Exclusion criteria

- Participants 0–2 years old or 19 years or older; studies where data could not be extracted for 3–18-year-olds.
- Pain assessed by proxy or with scales other than those included above.
- Case reports, case series, experimental studies of pain, and narrative reviews.
- Studies with fewer than 50 participants.

### Literature search strategy

Based on the PECO framework, search strategies were designed in collaboration with and implemented by an information specialist (MV). Records were identified through searches in PubMed via NLM, CINAHL via EBSCOhost, PsycINFO via EBSCO, and Web of Science Core Collection via Clarivate. The initial search was conducted in July 2022 and updated in February 2025, and finally in January 2026. No language restriction or other filters were applied. The reference lists of included studies were manually screened for additional records. No search for grey literature was performed. The search strategies are presented in Additional file 1. A search in PROSPERO revealed no ongoing or planned systematic reviews on the topic.

### Identification and screening

All abstracts were screened independently by eight reviewers working in pairs (RR/HD, HB/SP, GK/LP, SBR/LA) using Rayyan (https://rayyan.ai/). If at least one reviewer considered an abstract relevant, the full text was retrieved for further assessment. Assessment of the relevance of all full-text papers was conducted independently by four reviewers working in pairs (GK/HB, HD/RR). Any disagreements were resolved by discussions. Excluded full text publications are listed in Additional file 2. Before the screening started, 25 reports were randomly selected and used in a calibration training exercise attended by all readers. Calibration exercises, and training on the use of risk-of-bias assessment tools were also conducted with the researchers involved.

### Risk of bias assessment

The risk of bias assessment of the included papers was performed independently by at least two reviewers (GK and HB, HD and RR). Observational studies of exposure were assessed using a tool provided by the Swedish Agency for Health Technology Assessment and Assessment of Social Services (https://www.sbu.se/en/tools-to-assess-risk-of-bias/) (see Additional file 3), and ROBIS was used for systematic reviews [25]. The risk of bias was rated as high, moderate or low. Any disagreements were resolved through consensus discussions.

In the assessment of confounding, participant age and procedure severity or type were considered. For studies that included a wide age range, stratification into relevant age groups was required. In terms of procedure severity, studies were expected to either demonstrate comparability in procedure severity across groups or describe how differences were controlled for. Studies with a high risk of bias due to confounding were automatically classified as having an overall high risk of bias. An overall high risk of bias was also assigned if at least two other domains apart from confounding were judged to be at high risk. Studies assessed as having an overall moderate or low risk of bias were included in the review.

### Data extraction

Data extraction from studies with low or moderate risk of bias was performed by one author (RR) and audited by another (HB). Disagreements were resolved through consensus discussion. Items extracted included author, year of publication, country of origin, setting, procedure, study design, participant characteristics, exposure, exposure measure, outcome, outcome measure scale, outcome measure points, results, and statistical method. For two studies [26, 27], confirmation of the self-reported nature of the outcome was obtained by contacting the respective corresponding authors. In one study [28] the age stratification was unclear, but the authors did not respond to our inquiries.

### Method of analysis and rating the certainty of evidence

A Synthesis Without Meta-analysis (SWiM) was conducted for exposures investigated in at least two studies by outlining the results in Table 4. The certainty of evidence was assessed by four of the authors (RR, HD, HB, and GK) using GRADE (Grading of Recommendations Assessment, Development and Evaluation, http://www.gradeworkinggroup.org).

## Results

**Fig. 1:**
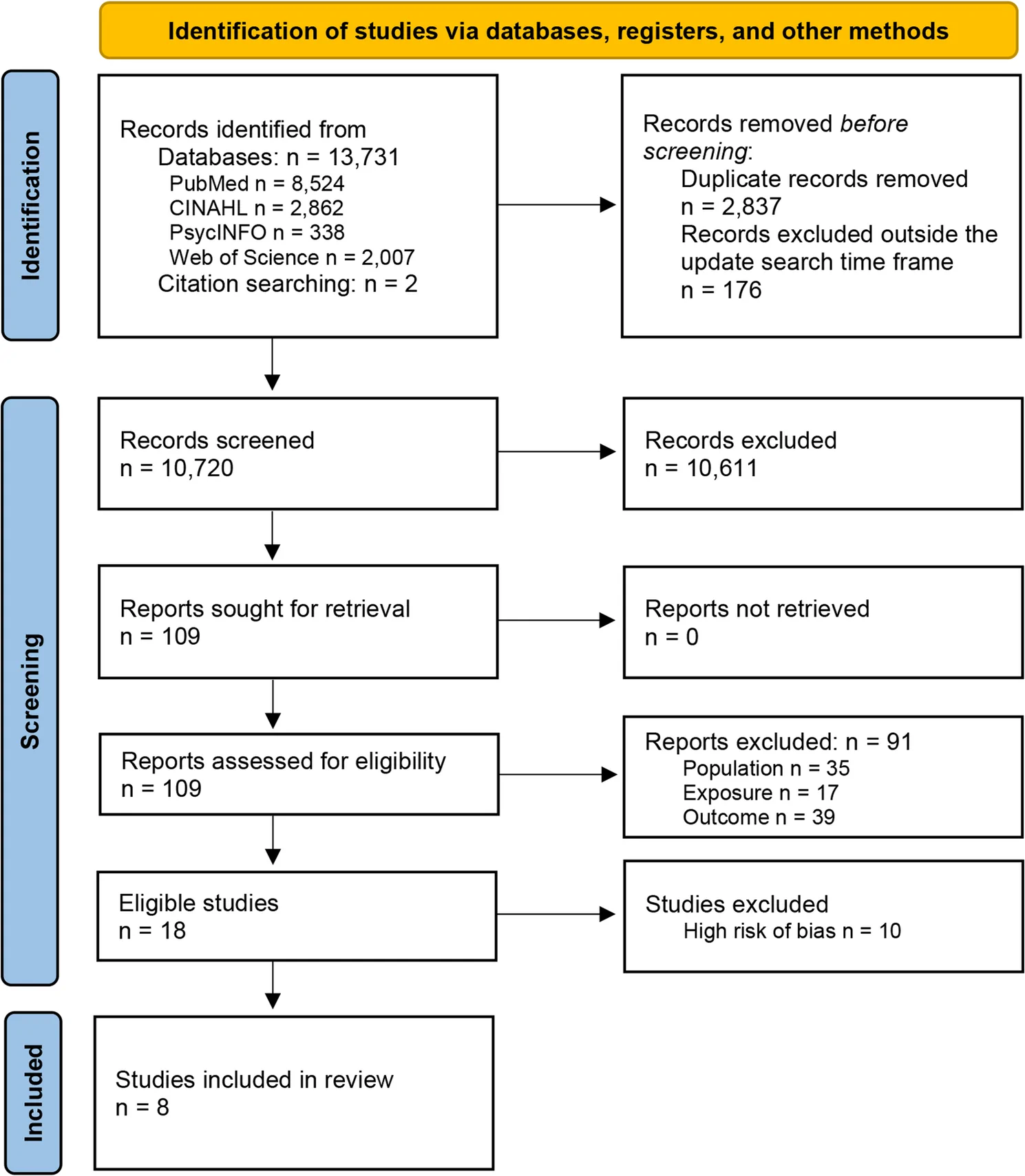
PRISMA flow diagram showing the literature review process

The systematic literature search identified 13,731 reports (Fig. 1). After duplicates were removed 10,720 records were screened (including two records identified through citation searching). In all, 109 full-text articles were retrieved and assessed for eligibility. Of these, 91 were excluded with reason (see Additional file 2), most often because the population characteristics or outcome measure did not meet the eligibility criteria.

The risk of bias was assessed for 17 primary studies (Tables 1 and 2) and one systematic review. The systematic review by Chieng et al. [29] was assessed as having a high risk of bias mainly due to high risk of bias in study identification and selection. Another potentially relevant systematic review was also identified [30], which examined sex‑related differences in self‑reported pain intensity during orthodontic treatment. However, this review included both paediatric and adult populations and did not specify outcome measures according to the PECO framework. Therefore, it was excluded during eligibility assessment. Among the 17 primary studies, nine were assessed as having a high risk of bias, often due to not controlling for confounding factors, such as differences in the treatments that the children received. Of the remaining eight studies, three were judged to have a low risk of bias and five a moderate risk. As the two studies by Bergius et al. [31, 32] included the same study population, data from these publications are presented together. Thus, eight studies based on seven populations were included in the review.

**Table 1:**
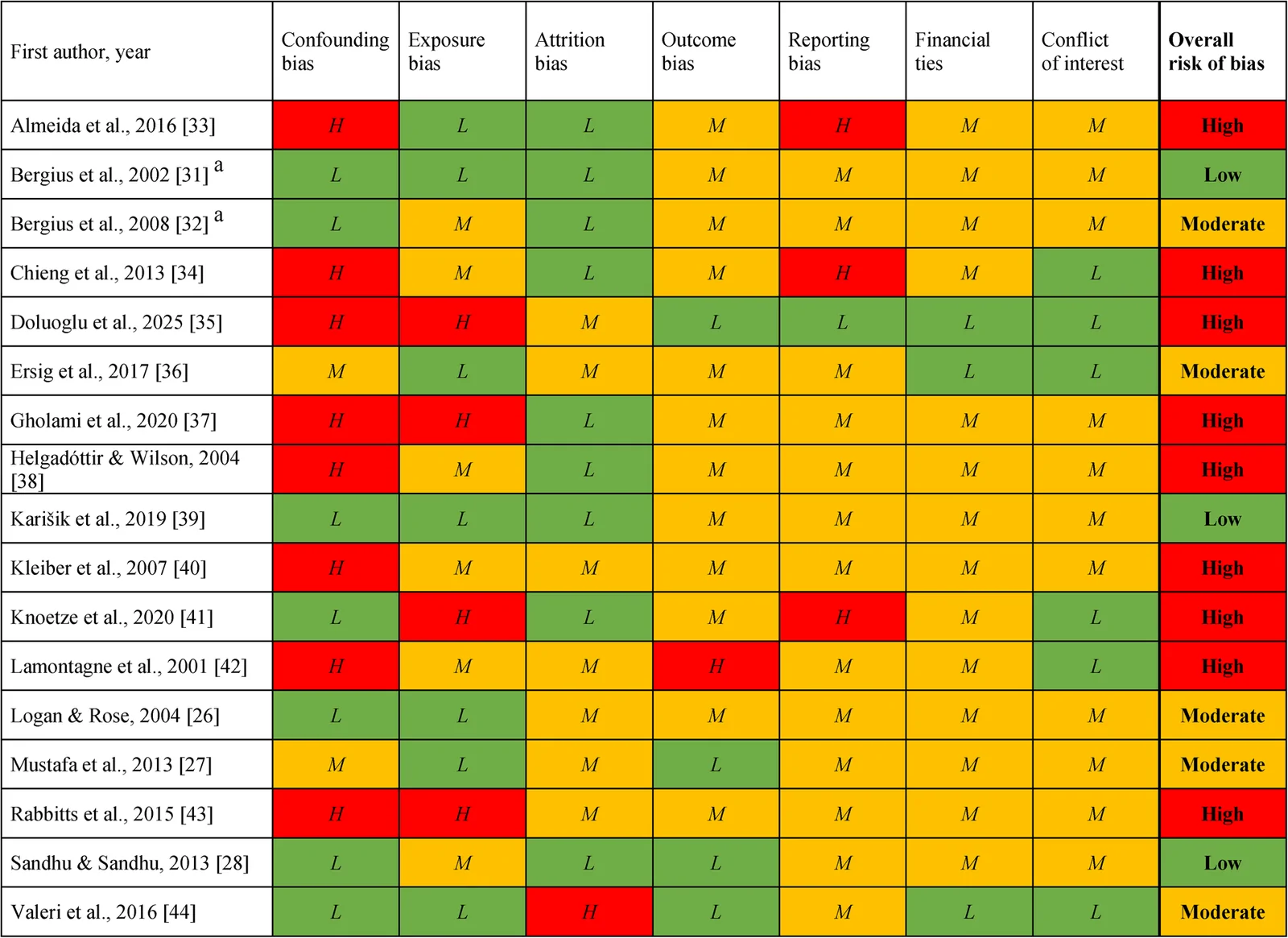
Risk of bias of eligible primary studies [26–28, 31–44]

*L* low risk of bias in green, *M* moderate risk of bias in yellow, *H* high risk of bias in red

^a^Two studies by Bergius et al. [31, 32] based on one study population

**Table 2:**
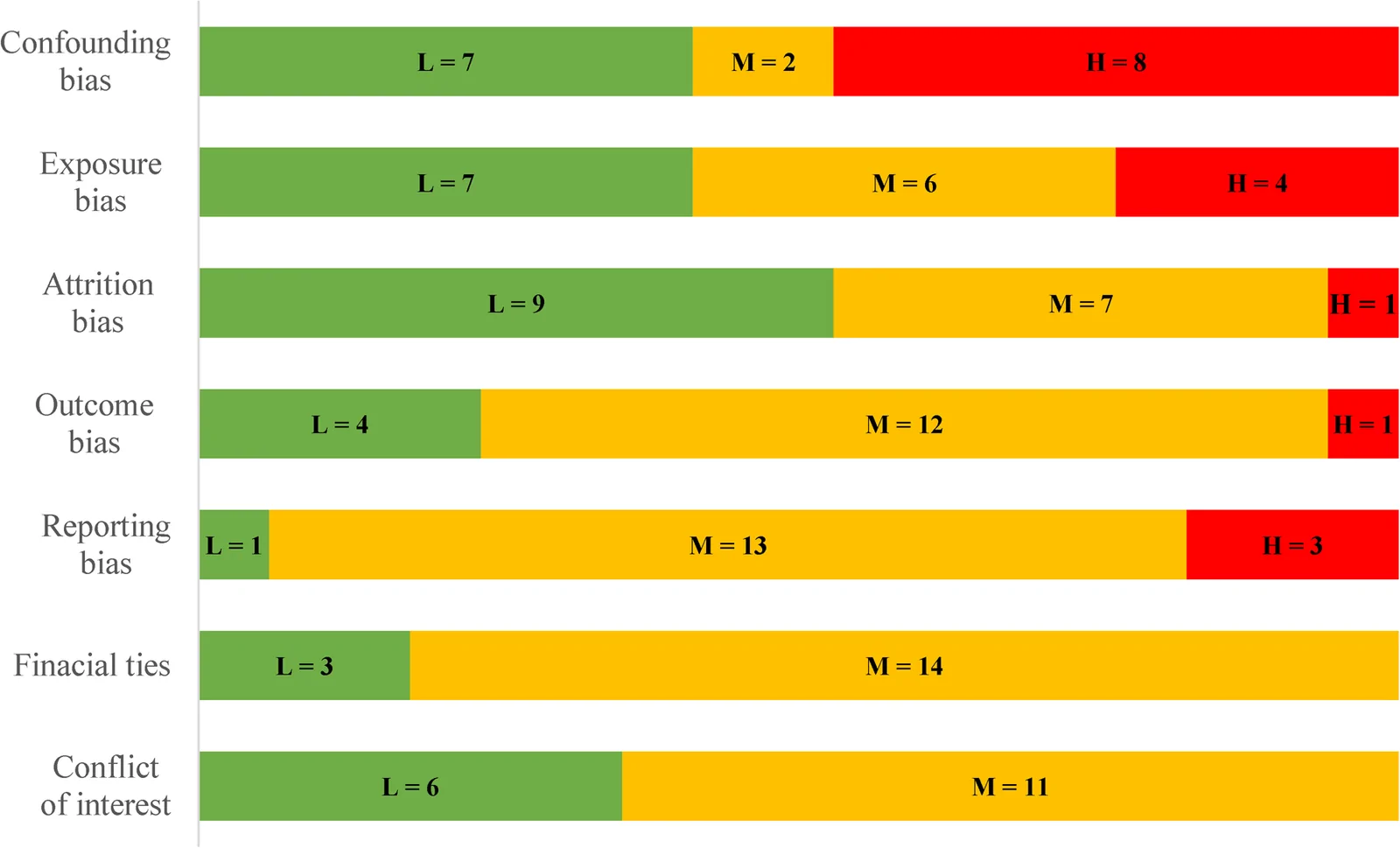
Risk of bias of eligible primary studies across the seven domains

Digits indicate the number of studies assessed as having: L low risk of bias in green, M moderate risk of bias in yellow, H high risk of bias in red

Note that there are two publications by Bergius et al. [31, 32] based on the same study population. The assessment for exposure bias differed between the two studies (Low and Moderate risk, respectively) and both publications are included in the table

The characteristics of the included studies and the extracted data are presented in Table 3. All included studies were of observational design and published between 2002 and 2019. These studies included a total of 1,508 participants (range 55–828), aged 3 to 18 years. Four studies were conducted in medical settings (1,048 participants, range 56–828, aged 3 to 18 years), one in Canada (venipuncture) [44], two in the USA (surgical procedures and venipuncture) [26, 36], and one in Montenegro (surgery for unilateral inguinal hernia) [39]. Four studies were conducted in dental settings (460 participants range, 55–280, aged 5 to 18 years), two Swedish studies based on the same population (orthodontic treatment) [31, 32], one Indian study (orthodontic treatment) [28], and one study from the UK (dental treatments) [27].

**Table 3:** Characteristics and extracted data of included studies with low or moderate risk of bias

First author Year Country	Study design Setting Procedure	Participants	Sex			Age			Other biological factors		
			Exposure	Outcome Scale Measure points	Results	Exposure	Outcome Scale Measure points	Results	Exposure	Outcome Scale Measure points	Results
Bergius 2002 [31] & 2008 [32] Sweden	Observational Dental Molar elastic separators bilaterally.	55 (32f/23m) 12–18 yrs (x̄ = 15.4 yrs)	Sex	Procedural pain (intensity) VAS^h^ Days 0, 1, 3, 5, 7	Mean pain higher for girls vs. boys on days 3 (*p* < 0.01), 5 (*p* < 0.001), and 7 (*p* < 0.05). Days 0 and 1, no significant differences. ^∆^	Age	Procedural pain (pain/no pain) VAS^h^ Days 0, 1, 3, 5, 7	Day 0: patients reporting pain older (r_s_ = 0.29; *P* < 0.05). Days 1–7 nonsignificant differences.^□^			
			Sex	Procedural pain (pain/no pain) VAS^h^ Days 0, 1, 3, 5, 7	Girls > boys reported pain on days 3 (*p* < 0.001), 5 (*p* < 0.001), and 7 (*p* < 0.05). Days 0 and 1 no significant differences.^∆^	Age	Procedural pain (pain/no pain) VAS^h^ Day 7	Same frequency of pain reports in younger (12–15 y.o.) and older age groups (16–18 y.o.).*			
			Sex	Procedural pain (pain/no pain) VAS^h^ Day 7	Girls > boys reported pain (chi^2^=4.0 *p* < 0.05).* Girls having an OR of 3.2 (C.I. 1.01–10.26) of reporting pain.^†^						
Ersig 2017 [36] USA	Observational Medical IV insertion	828 (411f/417m) 4–10 yrs (x̄ = 7.2, SD 1.9)							Single Nucleotide Poly-morphism Saliva sample	Procedural pain Oucher^k^ Directly after procedure	CRH gene (SNP rs12721510), associated with lower odds for high pain report (OR 0.570, C.I. 0.334–0.973, *p* = 0.039)^†^
											EDNRA gene (SNP rs10003447), associated with lower odds for high pain report (OR 0.741, C.I. 0.581–0.946, *p* = 0.016)^†^
											EDNRA gene (SNP rs4110), associated with higher odds for high pain report (OR 1.273, C.I. 1.008–1.608, *p* = 0.043)^†^
											EDNRA gene (SNP rs4110), associated with higher odds for high pain report (OR 1.273, C.I. 1.008–1.608, *p* = 0.043)^†^
											NKFB2 gene (SNP rs7897947), associated with lower odds for high pain report (OR 0.752, C.I. 0.570–0.993, *p* = 0.045)^†^
Karišik 2019 [39] Montenegro	Observational Medical Unilateral inguinal hernia repair	60 (30f/30m) 3–6 yrs (x̄ = 4.22)	Sex	Postoperative pain FACES^m^ 2, 4, 8, and 12 h after procedure	Males rate pain significantly higher than females at all time points.^◊^						
Logan 2004 [26] USA	Observational Medical Operative surgeries	104 (57f/47m) 12–18 yrs (x̄ = 15.0, SD 1.7)	Sex	Postoperative pain NRS^l^ Every 2 h, POD 0–2	Lowest reported pain: girls mean 2.8 (SD 1.7 significantly higher than boys mean 2.1 (SD 1.6). Average reported pain: girls mean 4.5 (SD 1.7) significantly higher than boys mean 3.7 (SD 1.8). Highest reported pain: girls mean 6.3 (SD 2.2) non-significantly higher than boys mean 5.6 (SD 2.3).^◊^						
Mustafa 2013 [27] UK	Observational Dental	125 (50f/75m) 5–17 yrs (x̄ = 10.5, SD 3.1)				Age	Postoperative pain FPS-R^i^ 5–7 y/o, VAS^h^ 7–18 y/o Postoperative hours 2, 4, and 6	Older patients significantly more frequently report pain at 4 and 6 h, but not at 2 h.^◊^			
Sandhu 2013 [28] India	Observational Dental Fixed orthodontic appliance	280 (138f/142m) 11–17 yrs (x̄ = 13.96, SD 2.01)	Sex	Procedural pain VAS^h^ Baseline,1, 4, 12, 24, 36, 48, 72, 120, and 168 h	14–17 y/o girls significantly higher reported pain than 14–17 y/o boys (t = 2.76; p = 0.0209) and 11–14 y/o boys (t = 2.91; p = 0.0206). No significant difference for pain reports between 11–14 y/o boys and girls ^◊^						
Valeri 2016 [44] Canada	Observational Medical Venipuncture	56 (33f/23m) (x̄ = 7.47 yrs, SD 0.33)							Gestational age (GA) at birth	Procedural pain CAS^j^ Directly after procedure	Higher GA significantly associated with higher pain ratings (β 0.38, C.I. 0.02–0.87, *p* = 0.04).^‡^

First author Year Country	Dental anxiety			Previous pain experiences			Psychological factors		
	Exposure	Outcome Scale Measure points	Results	Exposure	Outcome Scale Measure points	Results	Exposure	Outcome Scale Measure points	Results
Bergius 2002 [31] & 2008 [32] Sweden	Dental anxiety Dental Anxiety Scale (DAS)^a^	Procedural pain (pain/no pain) VAS^h^ Day 7	Pain group: Mean 7.9 Median 7.0 SD 3.2 No pain group: Mean 6.1 median 6.0 SD 2.3 Mann-Whitney U-value 242.0, *p* < 0.05.^∆^ Elevated level of dental fear associated with reporting pain (OR 6.4; C.I. 1.0-40.2).^†^	General pains VAS^h^	Procedural pain (intensity) VAS^h^ Days 0, 1, 3, 5, 7	Girls reported higher ratings of procedural pain on days 3 and 5 among those with high general pain scores (median cut-off, F = 4.0 and F = 6.9; both *P* < 0.05).^♦^	Motivation for treatment 5-step scale^g^	Procedural pain (pain/no pain) VAS^h^ Day 7	Low level of motivation associated with reporting pain (OR 6.5; C.I. 1.3–32.3).^†^
				Reporting vaccinations as painful VAS^h^	Procedural pain (pain/no pain) VAS^h^ Day 7	Ratings of pain from vaccination associated with reporting pain (OR 15.8; CI 2.5-102.3).^†^	Activity temperament (single dimension) Emotionality, Activity and Sociability Temperament Questionnaire (EAS)^b^	Procedural pain (pain/no pain) VAS^h^ Day 7	Low activity temperament (EAS) associated with reporting pain (OR 10.1; CI 1.1–93.9).^†^
Ersig 2017 [36] USA							Self-esteem ‘I think I am’^c^	Procedural pain (pain/no pain) VAS^h^ Day 7	Pain group: Mean 75.5 (SD 30.5). No pain group: Mean 78.0 (SD 23.1). Non-significant difference.^∆^
Karišik 2019 [39] Montenegro									
Logan 2004 [26] USA									
Mustafa 2013 [27] UK									
Sandhu 2013 [28] India	Dental Anxiety Modified Child Dental Anxiety Scale (faces) (MCDASf)^d^	Postoperative pain FPS-R^i^ 5–7 y/o, VAS^h^ 7–17 y/o Postoperative hours 2, 4, and 6	Higher MCDASf score (cut-off at 17) associated with higher pain scores at 2 h (*p* = 0.02) and 6 h (*p* = 0.03).						
Valeri 2016 [44] Canada									
				Number of neonatal invasive procedures	Procedural pain CAS^j^ Directly after procedure	Greater number of invasive procedures associated with higher pain ratings (β 0.44, C.I. 0.47–6.35, *p* = 0.02).^‡^	Parental Trait-Anxiety State-Trait Anxiety Inventory (STAI)^f^	Procedural pain CAS^j^ Directly after procedure	Higher level of parent Trait-Anxiety associated with higher pain ratings (β 0.38, C.I. 0.04–0.21, *p* = 0.007) in children.^‡^
				Number of neonatal surgeries		Lower number of neonatal surgeries associated with higher pain ratings (β − 0.32, C.I. −2.55-−0.23, *p* = 0.02).^‡^	Internalizing/Externalizing behaviour Child Behavior Checklist (CBCL)^e^		Lower CBCL-Externalizing scores associated with higher pain ratings (β − 0.30, C.I. −0.15-−0.009, *p* = 0.03).^‡^

^a^Corah NL. Development of a dental anxiety scale. J Dent Res. 1969 Jul-Aug;48(4):596. ^b^Buss AH, Plomin R. Temperament: early developing personality traits. Hillsdale, NJ: Lawrence Erlbaum Associates; 1984. ^c^Ouvinen-Birgerstam P. Jag tycker jag är; manual (I think I am: manual). Stockholm: Psykologiförlaget; 1985. ^d^Howard KE, Freeman R. Reliability and validity of a faces version of the Modified Child Dental Anxiety Scale. Int J Paediatr Dent. 2007 Jul;17(4):281-8. ^e^Achenbach, T.; Rescorla, L. Manual for the ASEBA preschool forms and profiles. Burlington, VT: University of Vermont, Research Center for Children, Youth, and Families; 2000. ^f^Spielberger CD. State-trait anxiety inventory for adults. 1983. Palo Alto (CA): Consulting Psychologists Press. ^g^Bergius M, Broberg AG, Hakeberg M, Berggren U. Prediction of prolonged pain experiences during orthodontic treatment. Am J Orthod Dentofacial Orthop. 2008 Mar;133(3):339.e1-8. ^h^VAS – Visual Analogue Scale [13]. ^i^FPS-R – Faces Pain Scale-Revised [18]. ^j^CAS – Coloured Analogue Scale [17]. ^k^Oucher Pain Scale [15]. ^l^NRS – Numeric Rating Scale [14]. ^m^Wong-Baker FACES© Pain Rating Scale [17]

^*^Chi-square, ^◊^Student’s t-test, ^∆^Mann-Whitney U-test, ^☐^Spearman’s Rank Correlation, ^†^Logistic regression, ^‡^Stepwise multiple regression, ^♦^Two-way ANOVA

Five studies investigated the effect of sex on reports of procedural [28, 31, 32] or postoperative pain [26, 39], involving 499 individuals divided across four distinct study populations. Karišik et al. [39] reported that males aged 3–6 years rated postoperative pain higher than females did. Conversely, studies involving adolescents reported that females rated pain higher than males [26, 28, 31, 32]. In one study these differences were only reported as statistically significant for low and moderate pain ratings [26]. Bergius et al. [31, 32] reported higher pain ratings among female than male patients at the later follow-up assessments (days 3–7), with pain subsiding more rapidly among male patients during the seven-day treatment period. Based on evidence from four studies [26, 28, 31, 32] the certainty of evidence for an association between female sex and higher ratings of procedural or postoperative pain in adolescents was judged to be low (Table 4). Karišik et al. [39] was not considered in the certainty assessment as its study population differed substantially in age distribution from those of the other studies, and reporting was insufficient to allow meaningful comparison.

Three studies [27, 31, 32] with a combined population of 180 children and adolescents reported on the association between age and pain experiences. Two studies based on the same study population of fifty-five 12–18-year-olds reported an association between older age and the occurrence of pain at the early assessment, but this difference was no longer evident at subsequent follow-up assessments [31, 32]. In contrast, findings from one study [27] including 125 children and adolescents across a broader age range (5–17 years) stated that older patients more frequently reported postoperative pain than younger patients after dental procedures. The certainty of evidence, based on results from two studies [27, 31], was rated as very low for an association between increasing age and rating pain higher (Table 4).

**Table 4:** Summary of findings and certainty of evidence for the exposures sex, age, and dental anxiety

Exposure	Number of participants (Number of studies) [reference]	Effect of exposure	Summary of results*	Certainty of evidence (GRADE)	Comment to the certainty of evidence
Sex	439 (4 studies) [26, 28, 31, 32]	Female adolescents (12–18 y.o.) rate procedural or postoperative pain higher than males	Higher pain ratings in females [28, 31, 32]. Lowest and average pain ratings higher in females [26].	⨁⨁◯◯	Indirectness − 1 (orthodontic treatment implies selected populations) Inconsistency − 1 (inconsistent findings in the different studies)
Age	180 (2 studies) [27, 31]	Older patients rate pain higher.	Older patients report higher pain day 0 [31]. Older patients report more pain [27].	⨁◯◯◯	Indirectness − 1 (skewed age distribution; orthodontic treatment implies selected population) Imprecision − 2 (unclear estimates, few individuals)
Dental Anxiety	180 (2 studies) [27, 32]	Children and adolescents reporting dental anxiety rate pain higher.	Higher dental anxiety associated with higher pain [27, 32].	⨁◯◯◯	Indirectness − 1 (differences in procedures and follow-up; orthodontic treatment implies selected population) Imprecision − 2 (unclear estimates, few individuals)

*For detailed results see Table 3

Dental anxiety was investigated in two studies involving 180 children and adolescents, aged 5–18 years [27, 32]. One study reported that elevated levels of dental anxiety were associated with reporting pain [27], while the other found an association with higher pain ratings [32]. However, based on these two studies, the certainty of evidence that children and adolescents with dental anxiety rate pain higher was assessed as very low (Table 4).

The ethical assessment in this paper is based on the framework by Heintz et al. [45] and adapted to a systematic review on exposure factors. The main ethical issues identified concern the theme *Effects on health* and the items *Health* (experiencing pain impact on quality of life) and *Knowledge gaps* (although both procedural and postoperative pains are common encounters in medical and dental care the present systematic review shows that there is limited knowledge concerning risk factors for experiencing pain). Issues also concern the theme *Compatibility with ethical norms* and the item autonomy (undergoing medical and dental procedures are seldom a choice for children and therefore respect for their autonomy and integrity is at risk during treatments). Assessing ethical vetting and informed consent procedures in the eight studies included in this systematic review revealed that five studies [26, 28, 31, 32, 44] declared both ethical approval and informed consent. One study reported ethical approval but nothing on consent [39]. Yet another study lacked information about ethical approval as well as informed consent [36]. Finally, one study described that after discussion with an ethics committee it was established that the study did not need ethical approval as it was regarded as a PROM study (Patient Reported Outcome Measure) [27].

## Discussion

This systematic review aimed at assessing factors influencing how children and adolescents experience procedural and postoperative pain in dental and medical contexts. Even though potential factors were suggested, the low number of included studies, often small sample sizes, differences in settings, and variation in exposure and outcome measures indicate that the results should be viewed with caution. However, the findings suggest that pain may have a multifactorial background.

This review was comprehensive in scope to ensure inclusion of relevant pain studies. We incorporated studies from both medical and dental contexts, study populations receiving a wide variety of treatments, and pain reports covering both postoperative and procedural pain. To our knowledge, this wide approach has not been presented before, and the review should be of interest to researchers and clinicians within both the medical and dental fields. The use of a PECO framework strengthened the review by providing a structured and transparent approach to defining the research question and eligibility criteria, and guiding the development of search strategies, ensuring that relevant studies are systematically identified and reducing the risk of selection bias. To minimise selection bias, the search strategy was deliberately broad to reduce the risk of overlooking studies relevant to the research question. Another strength of this review is the use of repeated calibration exercises and consensus discussions within the research team at each stage of the review process, ensuring consistency and rigour in study selection and data extraction. The research group’s extensive experience working with systematic reviews and collaboration with an information specialist further strengthened the process. Contacting authors for clarification on self-reporting was an additional strength.

To minimise the risk of selection bias, and to increase transparency, the review protocol was published in advance and adhered to throughout the research process, with two modifications. First, a minimum population size of 50 participants was introduced before screening commenced to reduce the risk of small-study effects as small study populations are prone to random errors and may inflate effect estimates and distort review results [46, 47]. Similar population size thresholds have been applied in an earlier systematic review [48]. This consideration was particularly relevant as the review included children and adolescents aged 3 to 18 years, a population characterised by substantial developmental differences in cognitive and communicative abilities, which may affect their capacity to understand and report an abstract and subjective experience such as pain. While the threshold was intended to improve the robustness of the evidence, it may also have introduced selection bias through the exclusion of smaller studies that otherwise met the eligibility criteria. Second, during the early stages of the review process, it became evident that pain was typically reported as a secondary outcome in studies evaluating the effectiveness of interventions, rather than being the primary focus. Therefore, intervention studies were excluded.

The inclusion of a wide range of exposures was made to minimise the risk of overlooking studies relevant to the research question. This was managed throughout the review process through continuous consensus discussions. A limitation was the small number of studies available for each exposure (one to five), potentially reflecting the ethical and clinical challenges of researching pain experiences in children. The study design of included studies posed further limitations, as all were observational, leaving the potential relationships between exposure and outcome open to confounding. Consequently, no causal inferences should be drawn. Several studies also had comparatively modest sample sizes, which may have left their findings less robust than would have been desired.

Proxy assessments by parents or healthcare professionals have demonstrated varying degrees of agreement with children’s self-reported pain assessments [49, 50]. However, as pain is a subjective and individual experience [1], self-report when available should be considered a primary source for assessing pain intensity in children [51]. Accordingly, validated and recommended self-report pain measures for children and adolescents were used as outcome measures in this systematic review [52]. This approach is a methodological strength but may have limited the number of included studies.

Limiting the assessment of the certainty of evidence to exposures that had been investigated in at least two studies was based on the fact that the rating of one single study would largely have been determined by the characteristics of that individual study and would potentially have given a misleading impression of a more comprehensive assessment of the evidence base. Furthermore, important GRADE domains, such as consistency of results across studies, are not possible to evaluate with only one study.

The Declaration of Helsinki states that research protocols ‘must be submitted for consideration, comment, guidance, and approval to the concerned research ethics committee before the research begins’ [53]. This principle has been incorporated in legislation and research ethics codes in many countries. Ethical approval from an independent review body is also advocated by the International Committee of Medical Journal Editors (ICMJE) (http://www.icmje.org/). Furthermore, the Council for International Organizations of Medical Sciences (CIOMS) and the World Health Organization (WHO) have published guidelines on ethical principles for research involving human participants [54], emphasising the importance of ethical review and informed consent. Against this background, it is problematic that not all studies included in this review reported assessment by research ethics committees or informed consent procedures. Lacking a declaration on ethical approval or informed consent does not necessarily indicate that these issues were not appropriately addressed. Still, given the importance of the Declaration of Helsinki, the CIOMS and WHO guidelines, and the ICMJE position on ethical conduct, scientific publications would be expected to provide information on ethical approval, preferably including a reference number, and describe how informed consent was obtained. Suggestions that deviations from these principles may be acceptable in older publications are unconvincing. The oldest study included in this review, published in 2002 [31], describes both ethical review and informed consent. Clear reporting of these aspects is an important quality indicator and should be included in all clinical research publications. Preferably, systematic reviews should also assess and report this. Most importantly, all clinical studies involving children should undergo ethical review and obtain informed consent from legal guardians, and, when appropriate, assent from the child depending on their age and maturity [53, 54].

As pain remains a widespread problem for children receiving care [4, 5, 55, 56], there is a need for dental and medical health care professionals not only to identify those in pain, but also to better understand the factors that may influence children’s experience of pain. This systematic review did not identify any specific factors that uniquely influence the pain experiences of children and adolescents. Given the limited evidence, this review highlights the need for more high-quality primary studies investigating exposure factors that may influence the experience of pain in children.

## Conclusions

The main finding of this systematic review was that the currently available evidence is limited, precluding firm conclusions about the influence of individual factors on children’s and adolescents’ experiences of self-reported pain in conjunction with dental or medical procedures. Although exposure factors such as age, sex and dental anxiety were identified across the included studies, methodological limitations restrict clear interpretation of their respective contributions. These findings underline the importance of recognising interindividual variation in children’s pain experiences and of avoiding assumptions about a child’s pain experience based on isolated characteristics. Rather, the results indicate that pain may be multifactorial and that influencing factors could interact with one another. Strengthening the evidence base on factors influencing children’s and adolescents’ pain experiences will require methodologically rigorous, high-quality studies involving broader age ranges and sufficiently large study populations. Future research should also improve the transparency and completeness of reporting on ethical review and informed consent procedures.

## Supplementary Information

Additional file 1.

Additional file 2.

Additional file 3.

## Data Availability

This systematic review is based on published data from published primary studies. See paper and Additional files for details.
