# Supplementary material for: Factors affecting the experience of procedural and postoperative pain in children and adolescents - a systematic review
Source: BMC Oral Health. 2026 Oct 1;26:1892. doi: 10.1186/s12903-026-10060-8 (PMC13632072; doi:10.1186/s12903-026-10060-8)
Supplement: Supplementary file 1 [file 12903_2026_10060_MOESM1_ESM.docx]

**Additional file 1.** Search strategies.

| **PubMed via NLM** | | |
| --- | --- | --- |
| Population | | |
| #1  Children age | "Child"[MeSH] OR "Adolescent"[MeSH] OR "Minors"[MeSH] OR "Students"[MeSH] OR "Puberty"[MeSH] OR child[Title/abstract] OR adolescent[Title/abstract] OR teen[Title/abstract] OR youth[Title/abstract] OR schoolchild[Title/abstract] OR juvenile[Title/abstract] OR preschool[Title/abstract] OR pediatric[Title/abstract] OR paediatric[Title/abstract] OR minor[Title/abstract] OR young[Title/abstract] OR student[Title/abstract] OR offspring[Title/abstract] OR pubert*[Title/abstract] OR school age[Title/abstract] OR boy[Title/abstract] OR girl[Title/abstract] | 5,042,832 |
| #2  Medical/surgical procedures | "Surgical Procedures, Operative"[MeSH] OR "Anesthesia, Local"[MeSH] OR surgery[Title/abstract] OR Local Anesthesia[Title/Abstract] OR Nerve Block[Title/Abstract] OR Operative Procedures[Title/Abstract] OR medical procedure[Title/Abstract] OR surgical procedure[Title/Abstract] | 4,630,662 |
| #3  Oral/dental procedures | "Surgery, Oral"[MeSH] OR "Oral Surgical Procedures"[Mesh] OR “Root Canal Therapy” [MeSH] OR “Dental Cavity Preparation”[MeSH] OR “Endodontics”[MeSH] OR “Pulpotomy”[MeSH] OR “Pulpectomy”[MeSH] OR “Dental Restoration, Permanent”[MeSH] OR “Dental Pulp Capping”[MeSH] OR “Tooth Extraction”[MeSH] OR “Dental Care”[MeSH] OR teeth extraction[Title/Abstract] OR tooth extraction[Title/Abstract] OR tooth removal[Title/Abstract] OR oral surgery[Title/Abstract] OR dental procedures[Title/Abstract] OR orthodontic procedures[Title/Abstract] OR orthodontic treatment[Title/Abstract] OR dental treatment[Title/Abstract] OR Dental Care[Title/abstract] OR Dental Treatment[Title/abstract] OR Pulp Capping[Title/abstract] OR Pulp Therapy[Title/abstract] OR Filling Therapy[Title/abstract] OR Dental Restoration[Title/abstract] OR Pulpectomy[Title/abstract] OR Pulpotomy[Title/abstract] OR Endodontic[Title/abstract] OR Cavity Prep*[Title/abstract] OR Root Canal[Title/abstract] | 233,467 |
| #4 | #2 OR #3 | 4,765,952 |
| Outcome | | |
| #5  Pain  procedural/  postoperative | "Pain, Postoperative"[MeSH] OR "Pain, Procedural"[Mesh] OR "Acute Pain"[MeSH] OR "Pain"[MeSH] OR Acute pain[Title/Abstract] OR procedural pain[Title/Abstract] OR post-operative pain[Title/Abstract] OR postoperative pain[Title/Abstract] OR post operative pain[Title/Abstract] OR post surgical pain[Title/Abstract] OR postsurgical pain[Title/Abstract] OR post-surgical pain[Title/Abstract] OR surgical pain[Title/Abstract] OR treatment related pain[Title/Abstract] OR ache[Title/Abstract] OR pain perception[Title/Abstract] | 550,061 |
| #6  Pain measurement/scale | "Pain Measurement"[Mesh] OR "Self Report"[Mesh] OR "Self-Assessment"[Mesh] OR pain measurement[Title/abstract] OR pain assessment[Title/abstract] OR pain scale[Title/abstract] OR pain report[Title/abstract] OR self report[Title/abstract] OR self assessment[Title/abstract] OR pain rating scale[Title/abstract] OR FPS-R[Title/abstract] OR color analog scale[Title/abstract] OR color analogue scale[Title/abstract] OR CAS[Title/abstract] OR visual analog scale[Title/abstract] OR visual analogue scale[Title/abstract] OR VAS[Title/abstract] OR wong baker faces pain rating scale[Title/abstract] OR wong baker faces[Title/abstract] OR numeric rating scale[Title/abstract] OR NRS[Title/abstract] OR Oucher[Title/abstract] OR poker chip[Title/abstract] OR color scale[Title/abstract] OR colour scale[Title/abstract] OR "pieces of Hurt" | 394,690 |
|  |  |  |
| Result | **#1 AND #4 AND #5 AND #6** | 8,524 |

| **CINAHL via EBSCOhost** | | |
| --- | --- | --- |
| Population | | |
| #1  Children age… | MH "Child+" OR MH "Minors (Legal)" OR MH "Adolescence+" OR MH "Students+" OR MH "Puberty+" OR AB(Child OR adolescent OR teen OR youth OR "school child" OR juvenile OR preschool OR pediatric OR paediatric OR minor OR minors OR young OR student OR offspring OR pubert* OR "school age" OR boy OR girl) OR TI( Child OR adolescent OR teen OR youth OR "school child" OR juvenile OR preschool OR pediatric OR paediatric OR minor OR minors OR young OR student OR offspring OR pubert* OR "school age" OR boy OR girl) | 1,775,174 |
| #2  Medical/surgical procedures | "Surgery, Operative+" OR MH "Anesthesia, Local" OR TI (surgery OR local anesthesia OR nerve block OR operative procedure OR medical procedures OR surgical procedure) OR AB(surgery OR local anesthesia OR nerve block OR operative procedure OR medical procedures OR surgical procedure) | 353,535 |
| #3  Oral/dental procedures | MH "Dental Care+" OR MH "Tooth Extraction" OR MH "Endodontics+" OR MH "Root Canal Therapy" OR TI ( Dental care OR teeth extraction OR tooth extraction OR tooth removal OR oral surgery OR dental procedure OR orthodontic procedure OR orthodontic treatment OR dental treatment OR pulp capping OR pulp therapy OR filling therapy OR dental restoration OR pulpectomy OR pulpotomy OR endodontic OR cavity prep* OR root canal ) OR AB ( Dental care OR teeth extraction OR tooth extraction OR tooth removal OR oral surgery OR dental procedure OR orthodontic procedure OR orthodontic treatment OR dental treatment OR pulp capping OR pulp therapy OR filling therapy OR dental restoration OR pulpectomy OR pulpotomy OR endodontic OR cavity prep* OR root canal ) | 51,576 |
| #4 | #2 OR #3 | 398,006 |
| Outcome | | |
| #5  Pain  procedural/  postoperative | MH "Pain+" OR MH "Pain, Procedural" OR MH "Postoperative Pain" OR TI ( acute pain OR procedural pain OR post-operative pain OR postoperative pain OR post operative pain OR post-surgical pain OR postsurgical pain OR post surgical pain OR surgical pain OR treatment related pain OR ache OR pain perception ) OR AB ( acute pain OR procedural pain OR post-operative pain OR postoperative pain OR post operative pain OR post-surgical pain OR postsurgical pain OR post surgical pain OR surgical pain OR treatment related pain OR ache OR pain perception ) | 268,030 |
| #6  Pain measurement/scale | MH "Self Report+" OR MH "Self Assessment" OR MH "Pain Measurement" OR MH "Wong-Baker FACES Pain Rating Scale" OR TI(Self report OR self assessment OR pain assessment OR pain scale OR pain report OR pain measurement OR pain rating OR pain management strateg*OR FACES Pain Rating Scale OR FPS OR FPS-R OR Color analog scale OR CAS OR visual analog scale OR VAS OR Wong Baker faces OR numeric rating scale OR NRS OR Oucher OR poker chip OR pieces of hurt OR color scale) OR AB(Self report OR self assessment OR pain assessment OR pain scale OR pain report OR pain measurement OR pain rating OR pain management strateg*OR FACES Pain Rating Scale OR FPS OR FPS-R OR Color analog scale OR CAS OR visual analog scale OR VAS OR Wong Baker faces OR numeric rating scale OR NRS OR Oucher OR poker chip OR pieces of hurt OR color scale) | 249,988 |
|  |  |  |
| Result | **#1 AND #4 AND #5 AND #6** | 2,862 |

| **PsycINFO via EBSCO** | | |
| --- | --- | --- |
| Population | | |
| #1  Children age… | TI ( Child* OR adolescent OR teen* OR youth OR school child* OR juvenile OR preschool OR pediatric* OR paediatric* OR minor OR minors OR young OR student OR offspring OR pubert* OR school age* OR boy* OR girl* OR pubescen*) ) OR AB ( Child* OR adolescent OR teen* OR youth OR school child* OR juvenile OR preschool OR pediatric* OR paediatric* OR minor OR minors OR young OR student OR offspring OR pubert* OR school age* OR boy* OR girl* OR pubescen*) ) | 1,777,153 |
| #2  Medical/surgical procedures | TI, TI ( surgery OR local anesthesia OR nerve block OR operative procedure* OR medical procedure* OR surgical procedure* ) OR AB ( surgery OR local anesthesia OR nerve block OR operative procedure* OR medical procedure* OR surgical procedure* ) | 40,648 |
| #3  Oral/dental procedures | TI ( teeth extraction* OR tooth extraction OR tooth removal OR oral surgery OR dental procedure* OR orthodontic procedure* OR orthodontic treatment* OR dental treatment* OR dental care OR pulp capping OR pulp therapy OR filling therapy OR dental restoration OR pulpectomy OR pulpotomy OR endodontic* OR cavity prep* OR root canal ) OR AB ( teeth extraction* OR tooth extraction OR tooth removal OR oral surgery OR dental procedure* OR orthodontic procedure* OR orthodontic treatment* OR dental treatment* OR dental care OR pulp capping OR pulp therapy OR filling therapy OR dental restoration OR pulpectomy OR pulpotomy OR endodontic* OR cavity prep* OR root canal ) | 2,574 |
| #4 | #2 OR #3 | 42,844 |
| Outcome | | |
| #5  Pain  procedural/  postoperative | TI ( pain OR ache ) OR AB ( pain OR ache ) | 119,066 |
| #6  Pain measurement/scale | TI ( pain measurement OR pain assessment OR pain scale OR pain report OR self assessment OR pain rating scale OR FPS-R OR color analog scale OR color analogue scale OR CAS OR visual analog scale OR visual analogue scale OR VAS OR Wong Baker faces pain rating scale OR Wong Baker faces OR numeric rating scale OR NRS OR Oucher OR poker chip* OR color scale OR colour scale OR "pieces of hurt" ) OR AB ( pain measurement OR pain assessment OR pain scale OR pain report OR self assessment OR pain rating scale OR FPS-R OR color analog scale OR color analogue scale OR CAS OR visual analog scale OR visual analogue scale OR VAS OR Wong Baker faces pain rating scale OR Wong Baker faces OR numeric rating scale OR NRS OR Oucher OR poker chip* OR color scale OR colour scale OR "pieces of hurt" ) | 51,687 |
|  |  |  |
| Result | **#1 AND #4 AND #5 AND #6** | 338 |

| **Web of Science Core Collection via Clarivate** | | |
| --- | --- | --- |
| Population | | |
| #1  Children age… | **TS=(Child* OR adolescent OR teen* OR youth OR "school child*" OR juvenile OR preschool OR pediatric* OR paediatric* OR minor OR minors OR young OR student OR offspring OR pubert* OR "school age*" OR boy* OR girl* OR pubescen*)** | 6,656,638 |
| #2  Medical/surgical procedures | **TS=(surgery OR "local anesthesia" OR "nerve block" OR "operative procedure*" OR "medical procedure*" OR "surgical proced)** | 1,937,765 |
| #3  Oral/dental procedures | **TS=("teeth extraction*" OR "tooth extraction" OR "tooth removal" OR "oral surgery" OR "dental procedure*" OR "orthodontic procedure*" OR "orthodontic treatment*" OR "dental treatment*" OR "dental care" OR "pulp capping" OR "pulp therapy" OR "filling therapy" OR "dental restoration" OR pulpectomy OR pulpotomy OR endodontic* OR "cavity prep*" OR "root canal")** | 97,165 |
| #4 | #2 OR #3 | 2,018,856 |
| Outcome | | |
| #5  Pain  procedural/  postoperative | **TS=("acute pain" OR "procedural pain" OR "post-operative pain" OR "postoperative pain" OR "post operative pain" OR "post-surgical pain" OR "postsurgical pain" OR "post surgical pain" OR "surgical pain" OR "treatment related pain" OR ache OR "pain perception" )** | 111,168 |
| #6  Pain measurement/scale | **TS=("pain measurement" OR "pain assessment" OR "pain scale" OR "pain report" OR "self assessment" OR "pain rating scale" OR FPS-R OR "color analog scale" OR "color analogue scale" OR CAS OR "visual analog scale" OR "visual analogue scale" OR VAS OR "Wong Baker faces pain rating scale" OR "Wong Baker faces" OR "numeric rating scale" OR NRS OR Oucher OR "poker chip*" OR "color scale" OR "colour scale" OR "pieces of hurt")** | 266,938 |
|  |  |  |
| Result | **#1 AND #4 AND #5 AND #6** | 2,007 |
