# Supplementary material for: Factors affecting the experience of procedural and postoperative pain in children and adolescents - a systematic review
Source: BMC Oral Health. 2026 Oct 1;26:1892. doi: 10.1186/s12903-026-10060-8 (PMC13632072; doi:10.1186/s12903-026-10060-8)
Supplement: Supplementary file 2 [file 12903_2026_10060_MOESM2_ESM.docx]

**Additional file 2.** List of excluded reports in full text, with reason.

| **#** | **First author** | **Year** | **Reason (PECO)** |
| --- | --- | --- | --- |
| 1 | Abbasi | 2025 | Outcome |
| 2 | Abu Alhaija | 2015 | Population |
| 3 | Alzubaidi | 2023 | Exposure |
| 4 | Asvanund | 2015 | Exposure |
| 5 | Azak | 2022 | Population |
| 6 | Bearden | 2002 | Outcome |
| 7 | Birnie | 2017 | Outcome |
| 8 | Blount | 2019 | Outcome |
| 9 | Bournaki | 1997 | Outcome |
| 10 | Broome | 1994 | Population |
| 11 | Bringuier | 2009 | Exposure |
| 12 | Carr | 1998 | Outcome |
| 13 | Caumo | 2000 | Outcome |
| 14 | Chavanes | 2022 | Exposure |
| 15 | Chieng | 2012 | Outcome |
| 16 | Collis | 2024 | Exposure |
| 17 | Crandall | 2009 | Outcome |
| 18 | Da | 2023 | Outcome |
| 19 | de Menezes Abreu | 2009 | Exposure |
| 20 | Dimova | 2013 | Outcome |
| 21 | Donaldson | 2020 | Outcome |
| 22 | Dudukucu | 2025 | Exposure |
| 23 | Elheeny | 2023 | Outcome |
| 24 | Einarsdóttir Egeland | 2023 | Population |
| 25 | Fan | 2018 | Population |
| 26 | Ferland | 2017 | Outcome |
| 27 | Fowler-Kerry | 1991 | Outcome |
| 28 | Franco Castanys | 2023 | Outcome |
| 29 | Gerbershagen | 2014 | Population |
| 30 | Gillies | 1999 | Exposure |
| 31 | Goodenough | 1999 | Outcome |
| 32 | Hay | 2009 | Population |
| 33 | Heli Kerimaa | 2023 | Exposure |
| 34 | Heli Kerimaa | 2023 | Outcome |
| 35 | Horn-Hofmann | 2018 | Population |
| 36 | Hu | 2025 | Exposure |
| 37 | Ileri | 2016 | Population |
| 38 | Jensen | 2012 | Exposure |
| 39 | Joestlein | 2015 | Outcome |
| 40 | Kay | 2021 | Population |
| 41 | Klages | 2006 | Population |
| 42 | Krekmanova | 2013 | Outcome |
| 43 | Kristensen | 2018 | Exposure |
| 44 | Kuscu | 2008 | Exposure |
| 45 | Lamarca | 2018 | Population |
| 46 | Lander | 1991 | Outcome |
| 47 | Lee | 1996 | Outcome |
| 48 | Levy | 2025 | Outcome |
| 49 | Lilley | 2000 | Outcome |
| 50 | Lindberg | 2020 | Population |
| 51 | Lorek | 2025 | Outcome |
| 52 | Marsac | 2010 | Outcome |
| 53 | Mathias | 2020 | Outcome |
| 54 | Moura | 2021 | Outcome |
| 55 | Moura-Leite | 2008 | Outcome |
| 56 | Mundim | 2019 | Outcome |
| 57 | Naoumova | 2012 | Outcome |
| 58 | Onwuka | 2020 | Population |
| 59 | Oommen | 2020 | Population |
| 60 | Pagé | 2013 | Outcome |
| 61 | Palermo | 1996 | Population |
| 62 | Palermo | 1998 | Population |
| 63 | Pavlić | 2025 | Exposure |
| 64 | Pavlin | 2005 | Population |
| 65 | Peretz | 2010 | Outcome |
| 66 | Puntillo | 2001 | Population |
| 67 | Rabbitts | 2015 | Outcome |
| 68 | Rheel | 2022 | Population |
| 69 | Rocha | 2009 | Population |
| 70 | Rosenbloom | 2025 | Outcome |
| 71 | Rullander | 2016 | Population |
| 72 | Salgado | 2022 | Population |
| 73 | Saloom | 2018 | Outcome |
| 74 | Sangalli | 2025 | Population |
| 75 | Schirmer | 2018 | Population |
| 76 | Schlenz | 2012 | Exposure |
| 77 | Segura-Egea | 2009 | Population |
| 78 | Sergl | 1998 | Population |
| 79 | Shaw | 2022 | Population |
| 80 | Stamer | 2021 | Exposure |
| 81 | Sujka | 2018 | Outcome |
| 82 | Takahashi | 2018 | Population |
| 83 | Topaloğlu | 2013 | Outcome |
| 84 | Üstünel | 2023 | Population |
| 85 | Valencia | 2022 | Population |
| 86 | Versloot | 2008 | Outcome |
| 87 | Vinall | 2013 | Population |
| 88 | Visoiu | 2024 | Exposure |
| 89 | von Baeyer | 2009 | Population |
| 90 | Wang | 2015 | Population |
| 91 | Zhamilov | 2023 | Population |

1. Abbasi H, Saini RS, Binduhayyim RIH, Kuruniyan MS, Mosaddad SA, Heboyan A. Comparison of behavioural, physiological, and self-reported pain responses in Pakistani children with dental pain: a descriptive cross-sectional study. Eur Arch Paediatr Dent. 2025 Dec;26(6):1181-1192. doi: 10.1007/s40368-025-01090-x.
2. Abu Alhaija ES, Abu Nabaa MA, Al Maaitah EF, Al-Omairi MK. Comparison of personality traits, attitude toward orthodontic treatment, and pain perception and experience before and after orthodontic treatment. Angle Orthod. 2015 May;85(3):474-9. doi: 10.2319/121813-927.1.
3. Alzubaidi MA, Drummond BK, Wu J, Jones A, Tahmassebi JF, Aggarwal VR. Predictors of Intra-Operative and Post-Operative Pain Associated with Routine Dental Procedures in Children: A Systematic Review and Meta-Analysis. Dent J (Basel). 2023 Dec 25;12(1):4. doi: 10.3390/dj12010004.
4. Asvanund Y, Mitrakul K, Juhong RO, Arunakul M. Effect of audiovisual eyeglasses during local anesthesia injections in 5- to 8-year-old children. Quintessence Int. 2015 Jun;46(6):513-21. doi: 10.3290/j.qi.a33932.
5. Azak M, Aksucu G, Çağlar S. The Effect of Parental Presence on Pain Levels of Children During Invasive Procedures: A Systematic Review. Pain Manag Nurs. 2022 Oct;23(5):682-688. doi: 10.1016/j.pmn.2022.03.011.
6. Bearden DJ, Feinstein A, Cohen LL. The influence of parent preprocedural anxiety on child procedural pain: mediation by child procedural anxiety. J Pediatr Psychol. 2012 Jul;37(6):680-6. doi: 10.1093/jpepsy/jss041.
7. Birnie KA, Chorney J, El-Hawary R; PORSCHE Study Group. Child and parent pain catastrophizing and pain from presurgery to 6 weeks postsurgery: examination of cross-sectional and longitudinal actor-partner effects. Pain. 2017 Oct;158(10):1886-1892. doi: 10.1097/j.pain.0000000000000976.
8. Blount RL. Commentary: Acute Pediatric Procedural Pain, Distress, and Coping. J Pediatr Psychol. 2019 Aug 1;44(7):798-802. doi: 10.1093/jpepsy/jsz041.
9. Bournaki MC. Correlates of pain-related responses to venipunctures in school-age children. Nurs Res. 1997 May-Jun;46(3):147-54. doi: 10.1097/00006199-199705000-00005.
10. Broome ME, Bates TA, Lillis PP, McGahee TW. Children's medical fears, coping behaviors, and pain perceptions during a lumbar puncture. Oncol Nurs Forum. 1990 May-Jun;17(3):361-7.
11. Bringuier S, Dadure C, Raux O, Dubois A, Picot MC, Capdevila X. The perioperative validity of the visual analog anxiety scale in children: a discriminant and useful instrument in routine clinical practice to optimize postoperative pain management. Anesth Analg. 2009 Sep;109(3):737-44. doi: 10.1213/ane.0b013e3181af00e4.
12. Carr TD, Lemanek KL, Armstrong FD. Pain and fear ratings: clinical implications of age and gender differences. J Pain Symptom Manage. 1998 May;15(5):305-13. doi: 10.1016/s0885-3924(97)00370-9.
13. Caumo W, Broenstrub JC, Fialho L, Petry SM, Brathwait O, Bandeira D, Loguercio A, Ferreira MB. Risk factors for postoperative anxiety in children. Acta Anaesthesiol Scand. 2000 Aug;44(7):782-9. doi: 10.1034/j.1399-6576.2000.440703.x.
14. Chavanes P, Benadiba J, Caci H, Poirée M. Effect of Child Position on Pain Experience During Lumbar Puncture: A Prospective Pilot Study. J Pediatr Hematol Oncol. 2022 Mar 1;44(2):e381-e385. doi: 10.1097/MPH.0000000000002232.
15. Chieng STJ, He HG, Chan SWC. Research in brief - An exploratory study of preoperative level of anxiety and postoperative pain in school-aged children undergoing surgery. Singapore Nursing J. 2012 Mar; 39(1):50-1.
16. Collis RW, Dry T, Chan G, Lim P, Oswald T. Sex related difference in postoperative pain and opioid use following posterior spinal fusion for adolescent idiopathic scoliosis. Spine Deform. 2024 May;12(3):711-715. doi: 10.1007/s43390-024-00826-x.
17. Crandall M, Lammers C, Senders C, Braun JV. Children's tonsillectomy experiences: influencing factors. J Child Health Care. 2009 Dec;13(4):308-21. doi: 10.1177/1367493509344821.
18. Da W. Preoperative Anxiety and Postoperative Pain in Pediatric Burn Patients and Their Correlation Analysis. Iran Red Crescent Med J. 2023 Feb;25(2):e254. doi: 10.32592/ircmj.2023.25.2.2548.
19. de Menezes Abreu DM, Leal SC, Frencken JE. Self-report of pain in children treated according to the atraumatic restorative treatment and the conventional restorative treatment--a pilot study. J Clin Pediatr Dent. 2009 Winter;34(2):151-5. doi: 10.17796/jcpd.34.2.9k67p786l7126263.
20. Dimova V, Horn C, Parthum A, Kunz M, Schöfer D, Carbon R, Griessinger N, Sittl R, Lautenbacher S. Does severe acute pain provoke lasting changes in attentional and emotional mechanisms of pain-related processing? A longitudinal study. Pain. 2013 Dec;154(12):2737-2744. doi: 10.1016/j.pain.2013.08.005.
21. Donaldson CD, Jenkins BN, Fortier MA, Phan MT, Tomaszewski DM, Yang S, Kain ZN. Parent responses to pediatric pain: The differential effects of ethnicity on opioid consumption. J Psychosom Res. 2020 Nov;138:110251. doi: 10.1016/j.jpsychores.2020.110251.
22. Dudukcu FT, Padir Z. Children's fear, pain, and anxiety before and after enema: A descriptive, cross-sectional study. J Pediatr Nurs. 2025 May-Jun;82:145-150. doi: 10.1016/j.pedn.2025.03.007.
23. Elheeny AAH, Sermani DI, Abdelmotelb MA. Postoperative pain perception and associated risk factors in children after continuous rotation versus reciprocating kinematics: A randomised prospective clinical trial. Aust Endod J. 2023 Sep;49 Suppl 1:345-352. doi: 10.1111/aej.12746.
24. Einarsdóttir Egeland S, Lie HC, Woldseth EM, Korsvold L, Ruud E, Larsen MH, Viktoria Mellblom A. Exploring reported distress before and pain during needle insertion into a venous access port in children with cancer. Scand J Caring Sci. 2023 Dec;37(4):927-937. doi: 10.1111/scs.13067.
25. Fan Q, Xie H, Ma Z, Chen Z, Yan T, Ge W. Perioperative predictors of moderate and severe postoperative pain in idiopathic scoliosis patients following spinal correction and fusion operations. Medicine (Baltimore). 2018 Nov;97(46):e13215. doi: 10.1097/MD.0000000000013215.
26. Ferland CE, Saran N, Valois T, Bote S, Chorney JM, Stone LS, Ouellet JA. Preoperative Distress Factors Predicting Postoperative Pain in Adolescents Undergoing Surgery: A Preliminary Study. J Pediatr Health Care. 2017 Jan-Feb;31(1):5-15. doi: 10.1016/j.pedhc.2015.12.008.
27. Fowler-Kerry S, Lander J. Assessment of sex differences in children's and adolescents' self-reported pain from venipuncture. J Pediatr Psychol. 1991 Dec;16(6):783-93. doi: 10.1093/jpepsy/16.6.783.
28. Franco Castanys T, Jiménez Carrión A, Ródenas Gómez F, Clemente García S, Melero Mascaray A, Janeiro Amela M, Busquets Bonet J. Effects of virtual tour on perioperative pediatric anxiety. Paediatr Anaesth. 2023 May;33(5):377-386. doi: 10.1111/pan.14639.
29. Gerbershagen HJ, Pogatzki-Zahn E, Aduckathil S, Peelen LM, Kappen TH, van Wijck AJ, Kalkman CJ, Meissner W. Procedure-specific risk factor analysis for the development of severe postoperative pain. Anesthesiology. 2014 May;120(5):1237-45. doi: 10.1097/ALN.0000000000000108.
30. Gillies ML, Smith LN, Parry-Jones WL. Postoperative pain assessment and management in adolescents. Pain. 1999 Feb;79(2-3):207-15. doi: 10.1016/s0304-3959(98)00178-x.
31. Goodenough B, Thomas W, Champion GD, Perrott D, Taplin JE, von Baeyer CL, Ziegler JB. Unravelling age effects and sex differences in needle pain: ratings of sensory intensity and unpleasantness of venipuncture pain by children and their parents. Pain. 1999 Mar;80(1-2):179-90. doi: 10.1016/s0304-3959(98)00201-2.
32. Hay I, Oates J, Giannini A, Berkowitz R, Rotenberg B. Pain perception of children undergoing nasendoscopy for investigation of voice and resonance disorders. J Voice. 2009 May;23(3):380-8. doi: 10.1016/j.jvoice.2007.10.008.
33. Kerimaa H, Hakala M, Haapea M, Vähänikkilä H, Serlo W, He HG, Pölkki T. Effectiveness of a Mobile App Intervention for Preparing Preschool Children and Parents for Day Surgery: Randomized Controlled Trial. J Med Internet Res. 2023 Sep 29;25:e46989. doi: 10.2196/46989.
34. Kerimaa H, Ruotsalainen H, Kyngäs H, Miettunen J, Pölkki T. Effectiveness of interventions used to prepare preschool children and their parents for day surgery: A systematic review and meta-analysis of randomised controlled trials. J Clin Nurs. 2023 May;32(9-10):1705-1722. doi: 10.1111/jocn.16156.
35. Horn-Hofmann C, Scheel J, Dimova V, Parthum A, Carbon R, Griessinger N, Sittl R, Lautenbacher S. Prediction of persistent post-operative pain: Pain-specific psychological variables compared with acute post-operative pain and general psychological variables. Eur J Pain. 2018 Jan;22(1):191-202. doi: 10.1002/ejp.1115.
36. Hu L, Hua Y, Wang L, Mao Z, Jia X, Lei Z, Chang D, Cheng W. Effect of Short-term Deep Breathing Exercises on Perioperative Anxiety and Pain in Pediatric Orthopedic Patients: A Randomized Controlled Trial. J Perianesth Nurs. 2025 Feb;40(1):69-75. doi: 10.1016/j.jopan.2024.03.009.
37. Ileri Z, Baka ZM, Akin M, Apiliogullari S, Basciftci FA. Effect of menstrual cycle on orthodontic pain perception : A controlled clinical trial. J Orofac Orthop. 2016 May;77(3):168-75. English. doi: 10.1007/s00056-016-0013-9.
38. Jensen B. Post-operative pain and pain management in children after dental extractions under general anaesthesia. Eur Arch Paediatr Dent. 2012 Jun;13(3):119-25. doi: 10.1007/BF03262857.
39. Joestlein L. Pain, Pain, Go Away! Evidence-Based Review of Developmentally Appropriate Pain Assessment for Children in a Postoperative Setting. Orthop Nurs. 2015 Sep-Oct;34(5):252-9; quiz 260-1. doi: 10.1097/NOR.0000000000000175.
40. Kay J, Simunovic N, Heels-Ansdell D, Bhandari M, Ayeni OR; FIRST Investigators. Lower body mass index and age are predictive of improved pain and health utility scores following arthroscopic management of femoroacetabular impingement. Knee Surg Sports Traumatol Arthrosc. 2021 May;29(5):1461-1473. doi: 10.1007/s00167-020-06395-6.
41. Klages U, Kianifard S, Ulusoy O, Wehrbein H. Anxiety sensitivity as predictor of pain in patients undergoing restorative dental procedures. Community Dent Oral Epidemiol. 2006 Apr;34(2):139-45. doi: 10.1111/j.1600-0528.2006.00265.x.
42. Krekmanova L, Hakeberg M, Robertson A, Klingberg G. Common experiences of pain in children and adolescents--an exploratory factor analysis of a questionnaire. Swed Dent J. 2013;37(1):31-8.
43. Kristensen HN, Lundbye-Christensen S, Haslund-Thomsen H, Graven-Nielsen T, Elgaard Sørensen E. Acute Procedural Pain in Children: Intervention With the Hospital Clown. Clin J Pain. 2018 Nov;34(11):1032-1038. doi: 10.1097/AJP.0000000000000625.
44. Kuscu OO, Akyuz S. Is it the injection device or the anxiety experienced that causes pain during dental local anaesthesia? Int J Paediatr Dent. 2008 Mar;18(2):139-45. doi: 10.1111/j.1365-263X.2007.00875.x.
45. Lamarca GA, Vettore MV, Monteiro da Silva AM. The Influence of Stress and Anxiety on the Expectation, Perception and Memory of Dental Pain in Schoolchildren. Dent J (Basel). 2018 Oct 22;6(4):60. doi: 10.3390/dj6040060.
46. Lander J, Fowler-Kerry S. Age differences in children's pain. Percept Mot Skills. 1991 Oct;73(2):415-8. doi: 10.2466/pms.1991.73.2.415.
47. Lee LW, White-Traut RC. The role of temperament in pediatric pain response. Issues Compr Pediatr Nurs. 1996 Jan-Mar;19(1):49-63. doi: 10.3109/01460869609026854.
48. Levy BB, Siu JM, Rosenbloom BN, Noel M, Chen T, Wolter NE. Parental anxiety and catastrophizing in pediatric tonsillectomy pain control: A multivariate analysis. Int J Pediatr Otorhinolaryngol. 2025 Mar;190:112244. doi: 10.1016/j.ijporl.2025.112244.
49. Lilley CM. Psychological Predictors of Children’s Pain and Parents’ Medication Practices Following Pediatric Surgery. University of British Columbia. 2000.
50. Lindberg M, Franklin O, Svensson J, Franklin KA. Postoperative pain after colorectal surgery. Int J Colorectal Dis. 2020 Jul;35(7):1265-1272. doi: 10.1007/s00384-020-03580-4.
51. Lorek M, Jarząbek A, Sycińska-Dziarnowska M, Gołąb S, Cichocka E, Spagnuolo G, Woźniak K, Szyszka-Sommerfeld L. Personality traits, pain perception, and patient attitudes toward orthodontic treatment with fixed appliances. Front Neurol. 2025 Mar 7;16:1547095. doi: 10.3389/fneur.2025.1547095.
52. Marsac ML, Funk JB. Relationships among psychological functioning, dental anxiety, pain perception, and coping in children and adolescents. J Dent Child (Chic). 2008 Sep-Dec;75(3):243-51.
53. Mathias FB, Cademartori MG, Goettems ML. Factors associated with children's perception of pain following dental treatment. Eur Arch Paediatr Dent. 2020 Feb;21(1):137-143. doi: 10.1007/s40368-019-00456-2.
54. Moura LA, Pereira LV, Minamisava R, Borges NC, Castral TC, Souza LAF. Severe acute postoperative pain self-reported by children after ambulatory surgeries: a cohort study. Rev Bras Enferm. 2021 Jun 11;74(suppl 4):e20200151. doi: 10.1590/0034-7167-2020-0151.
55. Moura-Leite FR, Ramos-Jorge ML, Bonanato K, Paiva SM, Vale MP, Pordeus IA. Prevalence, intensity and impact of dental pain in 5-year-old preschool children. Oral Health Prev Dent. 2008;6(4):295-301.
56. Mundim AP, Corrêa-Faria P, Costa LR. Do preschoolers with adverse birth outcomes have more distress during dental examination? Eur Arch Paediatr Dent. 2019 Dec;20(6):571-576. doi: 10.1007/s40368-019-00438-4.
57. Naoumova J, Kjellberg H, Kurol J, Mohlin B. Pain, discomfort, and use of analgesics following the extraction of primary canines in children with palatally displaced canines. Int J Paediatr Dent. 2012 Jan;22(1):17-26. doi: 10.1111/j.1365-263X.2011.01152.x.
58. Onwuka CI, Udeabor SE, Al-Hunaif AM, Al-Shehri WAK, Al-Sahman LA. Does preoperative dental anxiety play a role in postoperative pain perception after third molar surgery? Ann Afr Med. 2020 Oct-Dec;19(4):269-273. doi: 10.4103/aam.aam_68_19.
59. Oommen S, Shetty A. Does parental anxiety affect children's perception of pain during intravenous cannulation? Nurs Child Young People. 2020 May 6;32(3):21-24. doi: 10.7748/ncyp.2019.e1187.
60. Pagé MG, Stinson J, Campbell F, Isaac L, Katz J. Identification of pain-related psychological risk factors for the development and maintenance of pediatric chronic postsurgical pain. J Pain Res. 2013;6:167-80. doi: 10.2147/JPR.S40846.
61. Palermo TM, Drotar D. Prediction of children's postoperative pain: the role of presurgical expectations and anticipatory emotions. J Pediatr Psychol. 1996 Oct;21(5):683-98. doi: 10.1093/jpepsy/21.5.683.
62. Palermo TM, Drotar DD, Lambert S. Psychosocial predictors of children's postoperative pain. Clin Nurs Res. 1998 Aug;7(3):275-91. doi: 10.1177/105477389800700305.
63. Pavlić A, Blagec T, Meštrović S. Effect of verbal and written information on the perception of pain and analgesic consumption, in adolescent orthodontic patients: A randomised controlled trial. J Orthod. 2025 Mar;52(1):37-44. doi: 10.1177/14653125241264295. Epub 2024 Jul 27. PMID: 39066635.
64. Pavlin DJ, Sullivan MJ, Freund PR, Roesen K. Catastrophizing: a risk factor for postsurgical pain. Clin J Pain. 2005 Jan-Feb;21(1):83-90. doi: 10.1097/00002508-200501000-00010.
65. Peretz B, Sarnat H. Relations between dental and somatic pain in children. Int J Paediatr Dent. 2010 May;20(3):201-6. doi: 10.1111/j.1365-263X.2010.01041.x.
66. Puntillo KA, White C, Morris AB, Perdue ST, Stanik-Hutt J, Thompson CL, Wild LR. Patients' perceptions and responses to procedural pain: results from Thunder Project II. Am J Crit Care. 2001 Jul;10(4):238-51. PMID: 11432212.
67. Rabbitts JA, Zhou C, Groenewald CB, Durkin L, Palermo TM. Trajectories of postsurgical pain in children: risk factors and impact of late pain recovery on long-term health outcomes after major surgery. Pain. 2015 Nov;156(11):2383-2389. doi: 10.1097/j.pain.0000000000000281.
68. Rheel E, Malfliet A, Van Ryckeghem DML, Pas R, Vervoort T, Ickmans K. The Impact of Parental Presence on Their Children During Painful Medical Procedures: A Systematic Review. Pain Med. 2022 May 4;23(5):912-933. doi: 10.1093/pm/pnab264. PMID: 34453832.
69. Rocha EM, Marche TA, von Baeyer CL. Anxiety influences children's memory for procedural pain. Pain Res Manag. 2009 May-Jun;14(3):233-7. doi: 10.1155/2009/535941.
70. Rosenbloom BN, Frederiksen SD, Wang V, Park CS, Gordon G, Brar G, Rasic N, Stinson JN, Birnie KA, Rabbitts JA. Prognostic factors of chronic postsurgical pain in children and adolescents: a systematic review and meta-analysis. Reg Anesth Pain Med. 2025 Feb 5;50(2):144-152. doi: 10.1136/rapm-2024-105696.
71. Rullander AC, Lundström M, Lindkvist M, Hägglöf B, Lindh V. Stress symptoms among adolescents before and after scoliosis surgery: correlations with postoperative pain. J Clin Nurs. 2016 Apr;25(7-8):1086-94. doi: 10.1111/jocn.13137.
72. Salgado KR, Normando D, Brandão GAM. Is catastrophising associated with pain perception in early phase of orthodontic treatment? A cohort study. J Oral Rehabil. 2022 Mar;49(3):309-315. doi: 10.1111/joor.13279.
73. Saloom HF, Papageorgiou SN, Carpenter GH, Cobourne MT. The effect of obesity on orofacial pain during early orthodontic treatment with fixed appliances: a prospective cohort study. Eur J Orthod. 2018 Jul 27;40(4):343-349. doi: 10.1093/ejo/cjx064.
74. Sangalli L, Turek B, Herrero Babiloni A, Alessandri-Bonetti A, Robinson C, Savoldi F. Sex-related difference in self-reported pain intensity at early stages of orthodontic treatment with multibracket fixed appliances, clear aligners, and interproximal elastic separators: A systematic review and meta-analysis. Am J Orthod Dentofacial Orthop. 2025 Aug;168(2):130-158.e17. doi: 10.1016/j.ajodo.2025.02.018.
75. Schirmer C, Dos Santos GO, Rost JF, Ferreira MBC, Weidlich P. Factors associated with pain and analgesic consumption following non-surgical periodontal therapy under local anaesthesia and carried out by dental students. J Clin Periodontol. 2018 Jan;45(1):68-77. doi: 10.1111/jcpe.12833.
76. Schlenz AM, McClellan CB, Mark TR, McKelvy AD, Puffer E, Roberts CW, Sweitzer SM, Schatz JC. Sensitization to acute procedural pain in pediatric sickle cell disease: modulation by painful vaso-occlusive episodes, age, and endothelin-1. J Pain. 2012 Jul;13(7):656-65. doi: 10.1016/j.jpain.2012.04.001.
77. Segura-Egea JJ, Cisneros-Cabello R, Llamas-Carreras JM, Velasco-Ortega E. Pain associated with root canal treatment. Int Endod J. 2009 Jul;42(7):614-20. doi: 10.1111/j.1365-2591.2009.01562.x.
78. Sergl HG, Klages U, Zentner A. Pain and discomfort during orthodontic treatment: causative factors and effects on compliance. Am J Orthod Dentofacial Orthop. 1998 Dec;114(6):684-91. doi: 10.1016/s0889-5406(98)70201-x.
79. Shaw RE, Staffa SJ, Nasr VG, Tung C. Impact of Parental Primary Spoken Language on Postoperative Pain Management in Children, a Retrospective Cohort Study. Children (Basel). 2022 May 18;9(5):739. doi: 10.3390/children9050739.
80. Stamer UM, Bernhart K, Lehmann T, Setzer M, Stüber F, Komann M, Meissner W. 'Desire for more analgesic treatment': pain and patient-reported outcome after paediatric tonsillectomy and appendectomy. Br J Anaesth. 2021 Jun;126(6):1182-1191. doi: 10.1016/j.bja.2020.12.047.
81. Sujka J, St Peter S, Mueller CM. Do health beliefs affect pain perception after pectus excavatum repair? Pediatr Surg Int. 2018 Dec;34(12):1363-1367. doi: 10.1007/s00383-018-4354-x.
82. Takahashi K, Nishizawa D, Kasai S, Koukita Y, Fukuda KI, Ichinohe T, Ikeda K. Genome-wide association study identifies polymorphisms associated with the analgesic effect of fentanyl in the preoperative cold pressor-induced pain test. J Pharmacol Sci. 2018 Mar;136(3):107-113. doi: 10.1016/j.jphs.2018.02.002.
83. Topaloğlu N, Tekin M, Yıldırım S, Küçük A, Gönüllü B, Hancı V. Passive smoking increases pain perception in children undergoing venous catheterization. Acta Paediatr. 2013 Nov;102(11):e493-6. doi: 10.1111/apa.12358.
84. Üstünel F, Tura İ, Akçam AT, Erden S. The Effect of Preoperative Fear of Pain on Postoperative Pain Levels and the Amount of Analgesic Consumption. Pain Manag Nurs. 2023 Dec;24(6):617-621. doi: 10.1016/j.pmn.2023.04.015.
85. Valencia L, Becerra Á, Ojeda N, Domínguez A, Prados M, González-Martín JM, Rodríguez-Pérez A. Effect of Preoperative Anxiety on Postoperative Pain after Craniotomy. J Clin Med. 2022 Jan 22;11(3):556. doi: 10.3390/jcm11030556.
86. Versloot J, Veerkamp JSJ, Hoogstraten J. Children's self-reported pain at the dentist. Pain. 2008 Jul 15;137(2):389-394. doi: 10.1016/j.pain.2007.09.025.
87. Vinall J, Miller SP, Synnes AR, Grunau RE. Parent behaviors moderate the relationship between neonatal pain and internalizing behaviors at 18 months corrected age in children born very prematurely. Pain. 2013 Sep;154(9):1831-1839. doi: 10.1016/j.pain.2013.05.050.
88. Visoiu M, Chelly J, Sadhasivam S. Gaining Insight into Teenagers' Experiences of Pain after Laparoscopic Surgeries: A Prospective Study. Children (Basel). 2024 Apr 20;11(4):493. doi: 10.3390/children11040493.
89. von Baeyer CL, Spagrud LJ, McCormick JC, Choo E, Neville K, Connelly MA. Three new datasets supporting use of the Numerical Rating Scale (NRS-11) for children's self-reports of pain intensity. Pain. 2009 Jun;143(3):223-227. doi: 10.1016/j.pain.2009.03.002.
90. Wang J, Tang X, Shen Y, Shang G, Fang L, Wang R, Xu Y. The correlations between health-related quality of life changes and pain and anxiety in orthodontic patients in the initial stage of treatment. Biomed Res Int. 2015;2015:725913. doi: 10.1155/2015/725913.
91. Zhamilov V, Turgut A, Reisoglu A, Basa CD, Kacmaz İE, Kazimoglu C. Analysis of the factors affecting pain level during K-wire removal among pediatric elbow fractures. J Pediatr Orthop B. 2023 Mar 1;32(2):134-138. doi: 10.1097/BPB.0000000000001010.
