# Supplementary material for: Factors affecting the experience of procedural and postoperative pain in children and adolescents - a systematic review
Source: BMC Oral Health. 2026 Oct 1;26:1892. doi: 10.1186/s12903-026-10060-8 (PMC13632072; doi:10.1186/s12903-026-10060-8)
Supplement: Supplementary file 3 [file 12903_2026_10060_MOESM3_ESM.docx]

**S3.** Template for risk of bias assessment of exposure studies, Swedish Agency for Health Technology Assessment and Assessment of Social Services.

**Assessment of exposure studies**
**UPDATED 2021-09-22**

**Outcome:**

**Reference (title, author, year):**

**Reviewer:**

This review template should be seen as a basic template that can be adjusted to suit the research questions in the current project.

The project should already have considered risks of selection bias during the relevance assessment.

Important confounders as well as valid and reliable ways to measure them should be identified before the assessment (see manual).

For unadjusted measures (which need to be justified), or when the question of causality cannot/does not need to be answered, go directly to 1B.

**Overall risk of bias**

**Overall risk of systematic distortion of the results (risk of bias)**

☐ Low  ☐ Moderate  ☐ High  ☐ Unacceptably high

**If possible: what is the direction of the bias for this outcome?**

☐ Overestimates the effect of the exposure
☐ Underestimates the effect of the exposure

*Justification:*

**1. Bias from confounding**

**Risk of bias from confounding is judged as:**
☐ Low  ☐ High  ☐ Unclear  ☐ Unacceptably high

*Justification: see the questions below.*

**Do you judge that…?**

**1.1** The study has taken the project’s important confounders into account.

☐ Yes ☐ Probably yes ☐ Probably no ☐ No ☐ Information missing

If “No”, “Probably no” or “Information missing” on question 1.1, answer 1.2 to 1.4.

**1.2** The study’s confounders were measured using valid and reliable methods.

☐ Yes ☐ Probably yes ☐ Probably no ☐ No ☐ Information missing

**1.3** An appropriate analysis method was used to control for the study’s confounders.

☐ Yes ☐ Probably yes ☐ Probably no ☐ No ☐ Information missing

**1.4** Variations in exposure over time that may affect the outcome have been taken into account.

☐ Yes ☐ Probably yes ☐ Probably no ☐ No ☐ Information missing

**If possible: what is the direction of the bias for the outcome?**

☐ Overestimates the effect of the exposure
☐ Underestimates the effect of the exposure
☐ Cannot be judged

*Justification of the assessment:*

**2. Bias from exposure**

**Risk of bias from classification/definition of the exposure is judged as:**
☐ Low  ☐ High  ☐ Unclear  ☐ Unacceptably high

*Justification: see the questions below.*

**Do you judge that…?**

**2.1** The exposure groups were well defined.

☐ Yes ☐ Probably yes ☐ Probably no ☐ No ☐ Information missing

**2.2** The composition of the groups has not been influenced by knowledge of the outcome.

☐ Yes ☐ Probably yes ☐ Probably no ☐ No ☐ Information missing

**2.3** The exposure was measured using valid and reliable methods.

☐ Yes ☐ Probably yes ☐ Probably no ☐ No ☐ Information missing

**2.4** The exposure was well defined regarding dose (intensity, duration and frequency).

☐ Yes ☐ Probably yes ☐ Probably no ☐ No ☐ Information missing

**If possible: what is the direction of the bias for the outcome?**

☐ Overestimates the effect of the exposure
☐ Underestimates the effect of the exposure
☐ Cannot be judged

*Justification of the assessment:*

**3. Attrition (loss to follow-up)**

**Risk of bias from attrition is judged as:**
☐ Low  ☐ High  ☐ Unclear

*Justification: see the questions below.*

**Do you judge that…?**

**3.1** The attrition is acceptable.

☐ Yes ☐ Probably yes ☐ Probably no ☐ No ☐ Information missing

If “No” or “Probably no” on question 3.1, answer 3.2 and 3.3.

**3.2** Both the attrition and the reasons for attrition were similar between groups.

☐ Yes ☐ Probably yes ☐ Probably no ☐ No ☐ Information missing

**3.3** Appropriate methods have been used to take the attrition into account and to assess its impact.

☐ Yes ☐ Probably yes ☐ Probably no ☐ No ☐ Information missing

**If possible: what is the direction of the bias for the outcome?**

☐ Overestimates the effect of the exposure
☐ Underestimates the effect of the exposure
☐ Cannot be judged

*Justification of the assessment:*

**4. Measurement of the outcome**

**Risk of bias from measurement of the outcome is judged as:**
☐ Low  ☐ High  ☐ Unclear

*Justification: see the questions below.*

**Do you judge that…?**

**4.1** The outcome is measured using valid and reliable methods.

☐ Yes ☐ Probably yes ☐ Probably no ☐ No ☐ Information missing

**4.2** Data collection was the same across measurements/groups.

☐ Yes ☐ Probably yes ☐ Probably no ☐ No ☐ Information missing

**4.3** Those who measured the outcome were unaware of which exposure the participants had received.

☐ Yes ☐ Probably yes ☐ Probably no ☐ No ☐ Information missing

If “No” or “Probably no” on question 4.1, 4.2 or 4.3, answer 4.4.

**4.4** The results have not been distorted by this.

☐ Yes ☐ Probably yes ☐ Probably no ☐ No ☐ Information missing

**If possible: what is the direction of the bias for the outcome?**

☐ Overestimates the effect of the exposure
☐ Underestimates the effect of the exposure
☐ Cannot be judged

*Justification of the assessment:*

**5. Selective reporting**

**Risk of bias from selective reporting is judged as:**
☐ Low  ☐ High  ☐ Unclear

*Justification: see the questions below.*

**Do you judge that…?**

**5.1** The analyses were carried out according to a plan that was published before outcome data were available.

☐ Yes ☐ Probably yes ☐ Probably no ☐ No ☐ Information missing

**5.2** The reported results have *not* been selected from several ways of measuring the outcome (for example different scales or time points).

☐ Yes ☐ Probably yes ☐ Probably no ☐ No ☐ Information missing

**5.3** The reported results have *not* been selected from several different analyses of the same outcome.

☐ Yes ☐ Probably yes ☐ Probably no ☐ No ☐ Information missing

**If possible: what is the direction of the bias for the outcome?**

☐ Overestimates the effect of the exposure
☐ Underestimates the effect of the exposure
☐ Cannot be judged

*Justification of the assessment:*

**6. Conflicts of interest**

Do the authors report that they lack financial interests that may influence the results of the study?
☐ Yes ☐ No ☐ Information missing

Do the authors report that they lack other interests that may influence the results?
☐ Yes ☐ No ☐ Information missing

**If possible: what is the direction of the bias for the study’s results?**

☐ Overestimates the effect of the exposure
☐ Underestimates the effect of the exposure
☐ Cannot be judged

*Comment on conflicts of interest – for example project-specific circumstances:*
